# Facile synthesis of hollow spherical g-C_3_N_4_@LDH/NCQDs ternary nanostructure for multifunctional antibacterial and photodegradation activities

**DOI:** 10.1016/j.isci.2023.106213

**Published:** 2023-02-16

**Authors:** Leila Arjomandi-Behzad, Zeinab Alinejad, Mina Ranjbar Zandragh, Amir Golmohamadi, Hossein Vojoudi

**Affiliations:** 1 Faculty of Chemistry, Kharazmi University, Tehran, Iran; 2 College of Health Sciences, West Chester University of Pennsylvania, West Chester, PA, USA

**Keywords:** Materials synthesis, Nanomaterials, Photoabsorption

## Abstract

Heterojunction nanostructure construction and morphology engineering are considered to be effective approaches to improve photocatalytic performance. Herein, ternary hierarchical hollow structures consisting of cobalt-aluminum-layered double hydroxide (CoAl-LDH) nanoplates grown on hollow carbon nitride spheres (HCNS) and decorated with N-doped carbon quantum dots (NCQDs) were prepared using a templating method and a subsequent solvothermal process. The obtained HCNS@LDH/NCQD composites presented an improved performance in photocatalytic degradation of tetracycline and inactivation of *E. coli* compared with pure HCNS and LDH under visible light illumination. The enhanced photocatalytic activity of the designed photocatalyst could be attributed to the following reasons: (1) A special hollow structure provides more active sites and has multiple capabilities of light reflection by helping with a high specific surface area that improves the harvesting efficiency of solar light and (2) the strong synergistic effect among the constituents, which promotes separation and transfer of charge carriers and broadens the photo-response range.

## Highlights

• Novel LDH@N-HCS/CN photocatalyst was successfully fabricated
• LDH@N-HCS/CN demonstrated high photocatalytic activity for NP degradation
• The tailored photocatalyst showed excellent stability and reusability

## Graphical abstract

**Figure:**
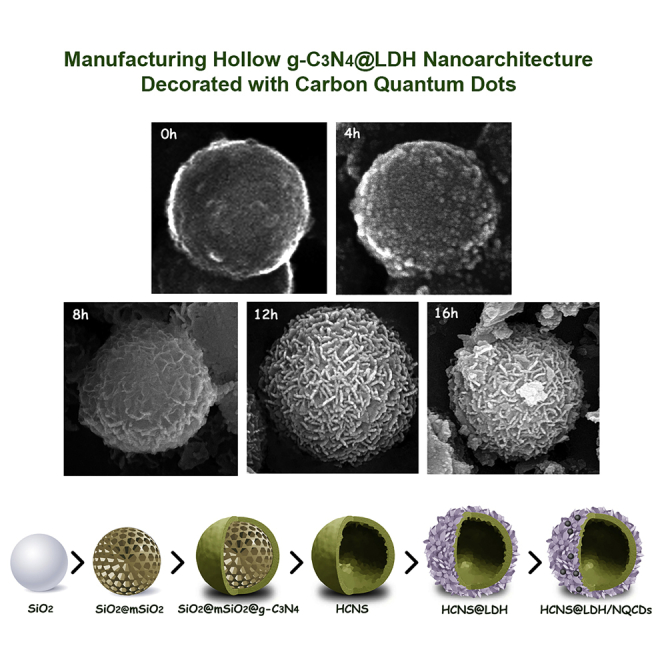

## Introduction

Nowadays, the rapid development of industries and an increasing world population have resulted in some issues, such as the energy crisis and environmental pollution.^1^^,^^2^^,^^3^^,^^4^^,^^5^ Organic pollutants, as an important part of environmental pollution, have become one of the hazardous threats to human health.^6^^,^^7^^,^^8^^,^^9^^,^^10^ Therefore, the development of green technology based on using renewable and clean energy resources has attracted attention to remove organic pollutants.^11^^,^^12^^,^^13^ It is widely accepted that photocatalysis based on semiconductor compounds is an advanced and promising technology to convert solar energy into chemical energy and decompose organic pollutants.^14^^,^^15^^,^^16^^,^^17^ The focus on practical photocatalytic applications is to develop efficient photocatalysts with a wide-spectrum response, low cost, and good durability.^18^^,^^19^ Hence, several types of photocatalysts, including metal oxides, metal chalcogenides, and oxynitrides, have been designed to improve the visible light response and quantum yield.^20^^,^^21^^,^^22^ Among the recently reported semiconductors, graphitic carbon nitride (g-C_3_N_4_) as a stable metal-free photocatalyst has received considerable attention in virtue of its moderate bandwidth of about 2.7 eV, low cost, facial synthesis, non-toxicity, and excellent thermal and chemical stability.^23^^,^^24^^,^^25^ Nevertheless, some disadvantages still limit the photocatalytic activity of pristine g-C_3_N_4_, for example, small specific surface area, low electrical conductivity, inadequate visible light utilization, and a high recombination rate of photo-generated electron-hole pairs.^26^^,^^27^^,^^28^ Various approaches, including heterojunction construction with other semiconductors, surface modification, metal or non-metal doping, and morphological synthesis, have been explored to overcome these shortcomings.^29^^,^^30^ Inspired by the morphology-dependent photocatalytic performance, many efforts have been devoted to changing the morphology of g-C_3_N_4_ to achieve higher photocatalytic performance.^19^^,^^31^^,^^32^ Thus, g-C_3_N_4_ with various morphologies, such as nanosheets, nanorods, nanotubes, and hollow spheres, have been reported.^33^^,^^34^^,^^35^ Among them, hollow spheres are more attractive in the photocatalysis field because of their unique structural properties, including large specific surface area, which supplies more active reaction centers and light scattering ability, which increases light utilization efficiency.^36^^,^^37^ Furthermore, hollow sphere structures can act as a support to assemble other semiconductors for designing core-shell heterojunctions. In principle, core-shell nanostructures with a larger contact area between the core and shell exhibit favorable charge separation efficiency.^38^

Recently, reports have demonstrated that layered double hydroxide (LDH) can be an attractive candidate for coupling with g-C_3_N_4_ to enhance the photocatalytic activity of pure carbon nitride.^39^ LDHs are a class of two-dimensional anionic clays constituted by positive metal ions in the sheets, which are uniformly surrounded by hydroxyl groups and oxo bridges, and interlayer anions to balance the charge of the sheets. Owing to their special layered structure and tunable composition, LDHs have many applications in the subject of photocatalysis. However, pure LDHs show poor photocatalytic performance because of the high recombination rate of photogenerated electron-hole pairs and the slow mobility of charge carriers.^40^ The rational design of the g-C_3_N_4_/LDH heterostructure can remove the limitations of pure LDHs and g-C_3_N_4_ and obtain an ideal photocatalyst with superior performance. Additionally, due to the high electron conductivity and electron reservoir properties, carbon dots (CDs), a new carbon nanomaterial type, can be used as an electron mediator between two semiconductors to decrease the recombination rate of electron-hole pairs.^41^^,^^42^ Furthermore, CDs can absorb light at longer wavelengths and convert it to shorter wavelengths through the up-conversion process. According to these properties, some studies have proven that the heterogeneous catalysts containing CDs exhibit favorable photocatalytic properties.^43^^,^^44^

Based on these considerations, we designed a core-shell structure of hollow carbon nitride spheres (HCNS) as a polymeric scaffold and Co-Al LDH modified with N-doped carbon quantum dots (NCQDs) as the shell for visible light photocatalytic application. Our strategy consists of the following three features. The first is the special morphology of the photocatalyst that provides a shorter charge diffusion pathway and multiple light reflections into the cavity for enhancing photocatalytic efficiency. The second point is a tight junction between the LDH shell and the g-C_3_N_4_ core for efficient separation of charge carriers. The last point is modifying the structure with NCQDs to decrease the recombination rate of charge carriers and increase the light absorption capacity. The photoactivity of this tailored structure was studied by the degradation of tetracycline (TC) as an antibiotic pollutant and the disinfection of *Escherichia coli* (*E. coli*) bacteria under visible light illumination. This present study can open new avenues to design tailored nanostructures for advanced photochemical applications.

## Results and discussion

### Synthesis and morphology characteristics

**Figure 1:**
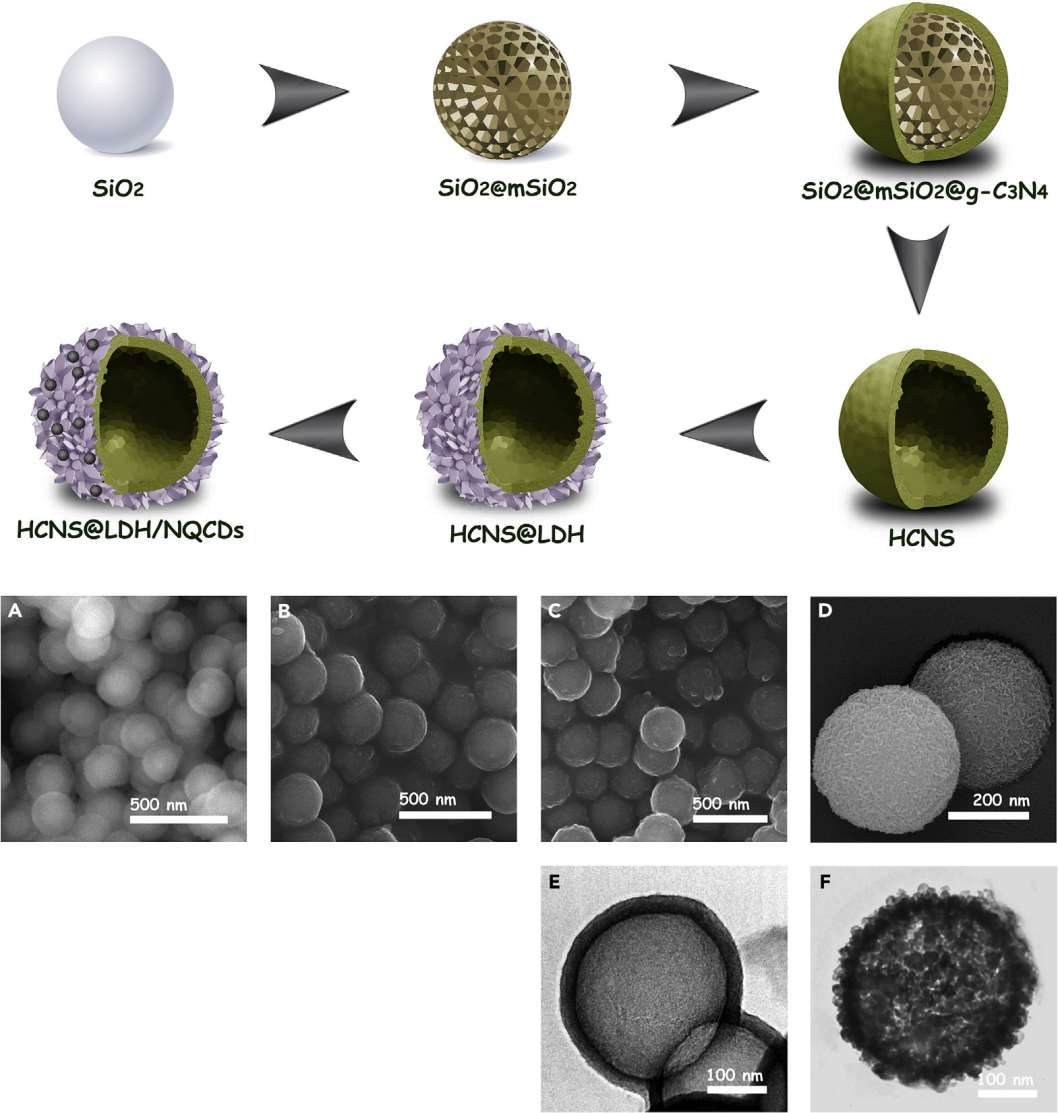
Flowchart diagram and Structural Characterization of the HCNS@LDH/NCQDs heterojunction (A) FESEM image of SiO_2_@mSiO_2_ showing uniform particles with solid core and porous shell. (B) FESEM image of SiO_2_@mSiO_2_@g-C_3_N_4_, confirming the formation of monodispersed spherical nanostructure. (C) FESEM image of HCNS, confirming the spherical shape of core-shell nanoparticles. (D) FESEM image of HCNS@LDH/NCQDs, confirming the growth of LDH nanoplates on the surface of carbon nitride. (E) TEM image of HCNS, confirming the hollow interior of carbon nitride. (F) TEM image of HCNS@LDH/NCQDs, confirming the hollow core-shell structure of the photocatalysts.

The hollow core-shell nano-photoreactor was prepared via a hard template strategy followed by a solvothermal process. Figure 1 shows the overall flowchart of the synthesis procedure and the corresponding field-emission scanning electron microscopy (FESEM) and transmission electron microscopy (TEM) images of the architectures at each production step. At first, the uniform silica nanospheres with a solid core and porous shell were synthesized and used as the sacrificed templates to synthesize the final structure. Cyanamide was loaded into the pores of the porous shell that was transformed into polymeric carbon nitride in the subsequent thermal polycondensation treatment (SiO_2_@mSiO_2_@g-C_3_N_4_). After removing the silica template, hydrothermal decorating of LDH/NCQDs on the surface of HCNS was performed to achieve the final architecture.

Figures 1A–1D show FESEM images at each step of the fabrication process. Figure 1A clearly exhibits that as-obtained silica templates have a uniform core-shell structure with a solid core and mesoporous shell and an average diameter of 250 nm. After the polycondensation of cyanamide into the mesoporous shell, SiO_2_@mSiO_2_@g-C_3_N_4_ particles were obtained, which had a monodispersed spherical nanostructure with an average diameter of 280 nm (Figure 1B). Figures 1C and 1E accordingly show the scanning electron microscopy and TEM images of HCNS after removing the silica template, respectively. The hollow interior of carbon nitride is confirmed by the TEM image, and the size of the core cavity was estimated at about 250 nm, which is about the size of silica nanosphere templates. The FESEM and TEM images of the HCNS@LDH/NCQDs are depicted in Figures 1D and 1F, respectively. The FESEM image shows that the LDH nanoplates grow uniformly on the carbon nitride. The TEM images reveal that the hollow core-shell structure comprises a hollow g-C_3_N_4_ substance and an ultrathin LDH shell. But NCQDs were not clearly visible in the FESEM and TEM images because of their tiny size, low content, and interference of LDH plates. Additionally, the X-ray elemental mapping analysis of the HCNS@LDH/NCQDs structure (Figure S1) confirms the dispersion of Co, Al, and O throughout the HCNS network.

**Figure 2:**
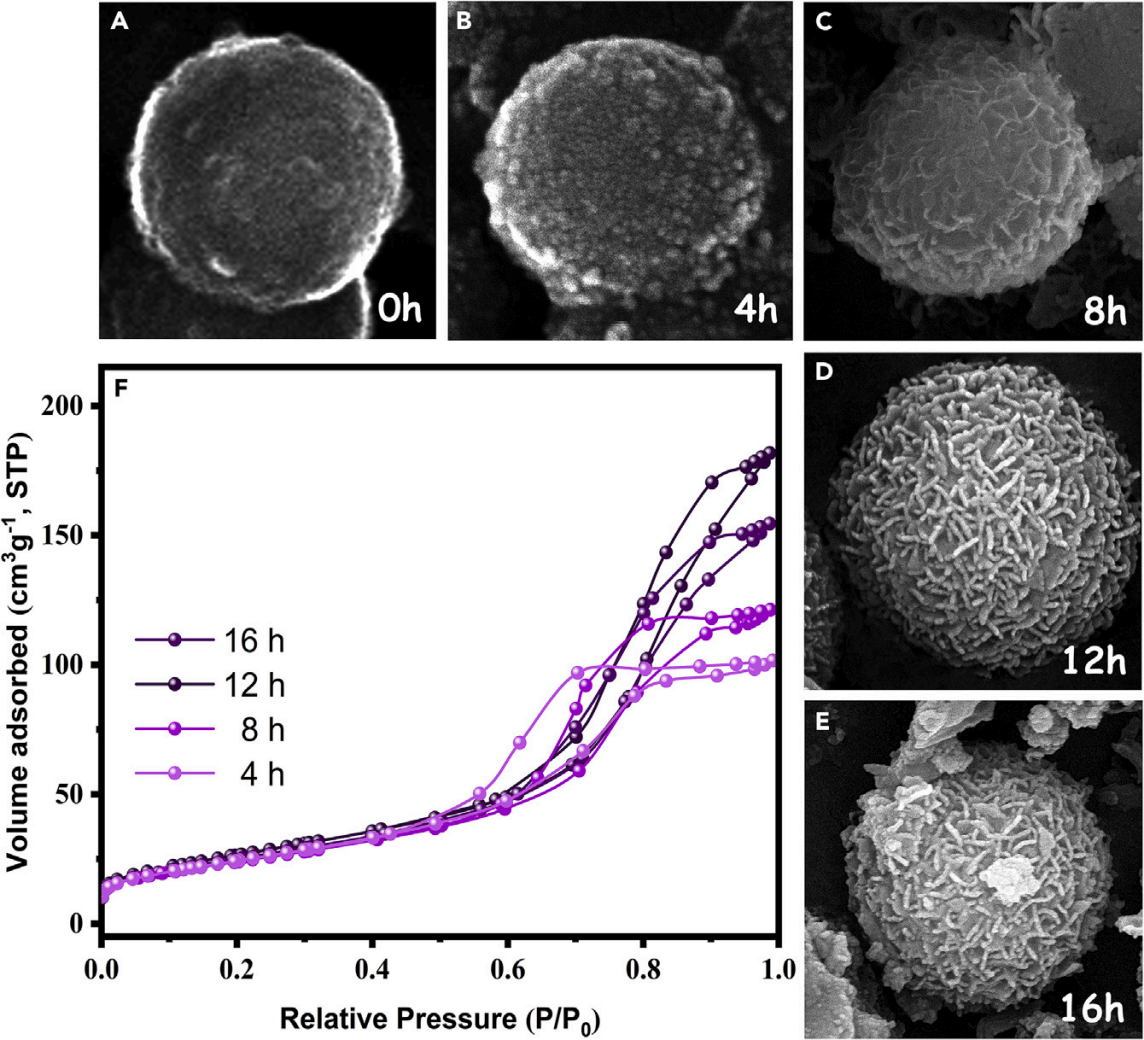
Time-dependent FESEM images and time-dependent N_2_ adsorption-desorption isotherm of as prepared samples in different reaction time (A–E) The FESEM images in different reaction time, showing the formation a uniform layer of LDH nanoplates on the surface of HCNS after 12 h of the reaction. (F) The porosimetry of prepared samples in different reaction time, indicating higher specific surface area of sample after 12 h of the reaction.

To provide insight into the formation process of the LDH shell on the surface of HCNS core, time-dependent growth experiments were carried out under the same conditions (Figure 2). The FESEM analysis reveals that under an early stage of 4 h, the surface of the HCNS is smooth (Figures 2A and 2B). As the hydrothermal process time was prolonged to 8 h, some nanoplates began to appear on the surface of the HCNS (Figure 2C). When the reaction time was further increased to 12 h, a hierarchical structure with a unique morphology was obtained (Figure 2D). However, by increasing the reaction time, the well-defined nanoplates started to agglomerate and disappeared in the sample grown for 16 h (Figure 2E). The specific surface area of these samples was analyzed by the N_2_ adsorption-desorption isotherms (Figure 2F). Among all four samples, the sample grown for 12 h showed the highest specific surface area, which may be attributed to more slit-shape pores formed by stacking LDH plates on the surface of the HCNS. According to the above results, it could be inferred that the hierarchical structure of the photocatalyst with well-defined morphology is favorable for providing more active sites, which can improve photocatalytic efficiency.

### Compositional and structural characterization

**Figure 3:**
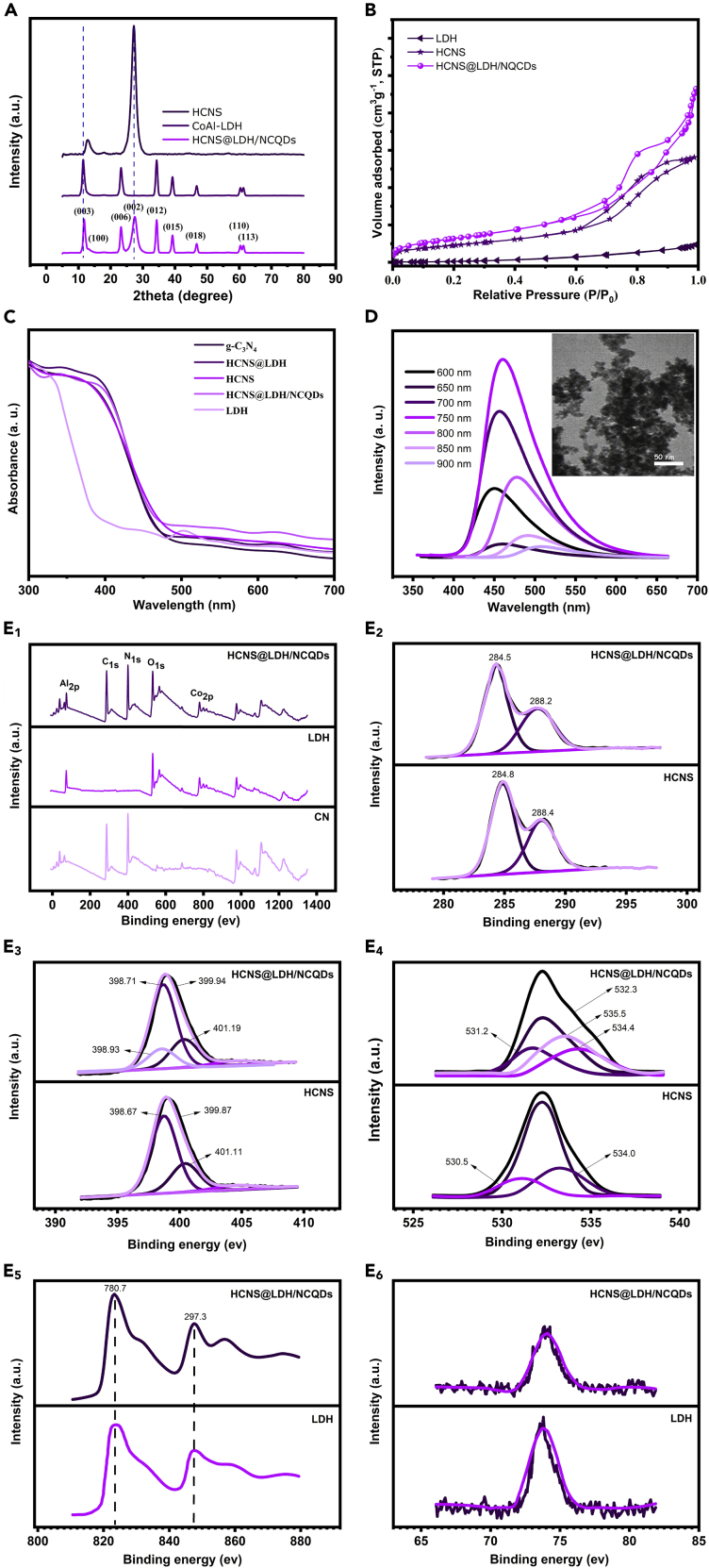
Structure characterization of as-synthesized samples (A) Powder XRD patterns of pure HCNS and LDH and ternary composite. (B) Nitrogen adsorption-desorption isotherms of HCNS, LDH, and HCNS@LDH/NCQDs. (C) UV-vis absorbance spectra of the as-prepared samples. (D) Upconverted photoluminescence spectra of the NCQDs and corresponding TEM image (inset). (E) (3E_1_) XPS survey spectra of HCNSN, Co-Al LDH, and HCNS@LDH/NCQDs; (3E_2_) high-resolution C 1s of HCNS and HCNS@LDH/NCQDs; (3E_3_) the high-resolution N 1s spectra of HCNS and HCNS@LDH/NCQDs; (3E_4_) the high-resolution O 1s of HCNS and HCNS@LDH/NCQDs; (3E_5_) the high-resolution Co 2p of LDH and HCNS@LDH/NCQDs; and (3E_6_) the high-resolution Al 2p of LDH and HCNS@LDH/NCQDs.

The crystal structure of the prepared samples was examined by X-ray diffraction (XRD), and the results are presented in Figure 3A. As shown in the figure, both HCNS and HCNS@LDH/NCQDs exhibit two distinct diffraction peaks at 13.21 and 27.28, corresponding to the (100) and (002) planes. The first peak belongs to the in-plane reduplicative of N-bridged tri-*s*-triazine units, and the second one belongs to the interlayer stacking of the conjugated aromatic systems. This result reveals that the crystal structure of HCNS is preserved during the hydrothermal decoration of 2D CoAl-LDH plates on the surface. Additionally, the diffraction value of (200) plane shows a slight shift in the case of the composite compared with pristine HCNS, suggesting the strong coordination between components in the HCNS@LDH/NCQDs composite. For pure CoAl-LDH, diffraction peaks at 2θ values of 12.1°, 24.3°, 35.9°, 39.1°, 47.2°, 61.2°, and 62.3° are ascribed to the (003), (006), (009), (012), (018), (110), and (113) planes, which indicates the hydrotalcite phase with CO_3_^2−^ intercalating in the interlayer spaces.^40^^,^^45^ In the heterostructure photocatalyst, superimposition of XRD patterns of CoAl-LDH and HCNS can be observed, which verifies that the LDH plates are successfully anchored on the surface of HCNS. Besides, the slight shift of (003) diffraction peak exists in the composite, which can reveal that some NCQDs intercalate into the interlayer space of CoAl-LDH nanosheets. Moreover, no diffraction peak for NCQDs was observed owing to their poor crystallinity and the small amount in the composite.

The porosity is a key factor for photoactive materials in photocatalytic applications. The N_2_ adsorption-desorption was performed to analyze the porous structure of the as-synthesized samples. As shown in Figure 3B, the isotherms of three samples, including HCNS and HCNS@LDH/NCQDs, could be regarded as type IV with distinct hysteresis loops, indicating both these samples have a mesoporous structure. HCNS@LDH/NCQDs show notable hysteresis loops without any limiting adsorption at the high P/P_0_ range, indicating the existence of slit-shape pores in the samples. The porosity properties of the samples consisting of specific surface area (S_BET_), average pore size (D_BJH_), and total pore volume are listed in Table S1. Thus, the high surface area and porous features of hierarchical HCNS@LDH/NCQDs can provide a unique opportunity to boost photocatalytic activity to a new level. The light absorption ability of the catalyst plays a vital role in the photocatalytic process. In this regard, the optical properties of HCNS, HCNS@LDH, and HCNS@LDH/NCQDs, as well as pure g-C_3_N_4_ and CoAl-LDH, were characterized by the UV-Vis diffuse reflection spectra. As shown in Figure 3C, pristine g-C_3_N_4_ shows an intrinsic absorption edge at around 475 nm, indicating its visible light absorption characteristics. As expected, HCNS has an extended absorption ability compared to the pristine g-C_3_N_4_, which is corroborated by the multiple reflections of light into the cavity. The LDH shows two distinct absorption features, including ligand-to-metal charge transfer in the UV range (200–300 nm) and three bands located in the visible region (450–530 nm) that can be attributed to the d-d transitions of Co^2+^ in octahedral coordination.^40^ Compared with HCNS@LDH, the HCNS@LDH/NCQDs sample exhibits an improvement in the visible light absorption, confirming that NCQDs successfully settle onto the surface of LDH nanoplates and change the optical properties of obtained photocatalyst.

The morphological and optical properties of the NCQDs were probed by TEM and photoluminescence (PL) analyses. The optical property of NCQDs was analyzed by photoluminescent spectroscopy. As shown in Figure 3D, NCQDs can serve as an intermedium that absorbs light of longer wavelengths in the visible and near-infrared regions and then emits visible light with shorter wavelengths.^46^ This outcome reveals that NCQDs can increase the light usage of the nanocomposite through the up-conversion process. Therefore, HCNS@LDH/NCQDs composite with extended visible light absorption is promising for boosting the visible-light-driven photocatalytic reactions. Furthermore, the TEM image of the prepared NCQDs is illustrated in the inset of Figure 3D. As can be seen, NCQDs are well dispersed with a uniform size and a quasi-spherical shape.

X-ray photoelectron spectroscopy (XPS) analysis was performed to probe the surface chemical composition and chemical states of the elements in the as-prepared samples. The survey spectrum of HCNS indicates the coexistence of C and N; the survey spectrum of CoAl-LDH shows the existence of Co, Al, and O; and the survey spectrum of HCNS@LDH/NCQDs shows the existence of C, N, O, Co, and Al without other impurity elements (Figure 3E_1_). As shown in Figure 3E_2_, the high-resolution XPS peaks of HCNS (C 1s) could be resolved into two peaks located at 284 and 288 eV, which are related to C-C bonds in graphitic carbon and sp^2^-bonded carbon in the s-triazine species, respectively. Compared with the pristine HCNS, the intensity of the C-C bond is higher than that for HCNS@LDH/NCQDs, which can be attributed to the successful incorporation of NCQDs. Additionally, a slight decrease in the binding energy of C 1s was observed compared to the pristine HCNS. The N 1s spectrum of the HCNS sample can be deconvoluted into the three main peaks that appeared in 401.11, 399.87, and 398.67 eV, which are assigned to the sp^2^ hybridized aromatic nitrogen in the triazine ring (C=N-C), the tertiary nitrogen groups (N-(C)_3_), and the amino functional groups (C-N-H), respectively (Figure 3E_3_). However, the binding energies of N 1s for HCNS@LDH/NCQDs are in good accordance with HCNS, and no shifts were observed in the heterostructure sample. Meanwhile, the N 1s spectrum of the heterojunction photocatalyst shows up to shift with another extra peak at 398.93 eV, corresponding to the Co-N bond in the composite. This extra bond approves that the ternary photocatalyst is not a simple physical mixture but a heterostructure with strong chemical interactions between the components. The Co 2p spectrum (Figure 3E_5_) for pure CoAl-LDH consists of two peaks at 780.6 and 297.2 eV, corresponding to Co 2p_1/2_ and Co 2p_3/2_, respectively. However, the binding energies of Co 2p have a shift toward higher values for the heterojunction photocatalysts than for pure LDH. The upshifts of Co 2p and N 1s peaks and shift C 1s in the lower binding energy suggest the chemical and environmental change arising from the interfacial interaction between HCNS, LDH, and NCQDs components in the heterostructure, which is beneficial for the migration of charge carriers. Besides, the high-resolution Al 2p spectrum reveals the presence of the Al^3+^ species in both pure LDH plates and ternary photocatalysts (Figure 3E_6_).

### Photocatalytic activity evolution

**Figure 4:**
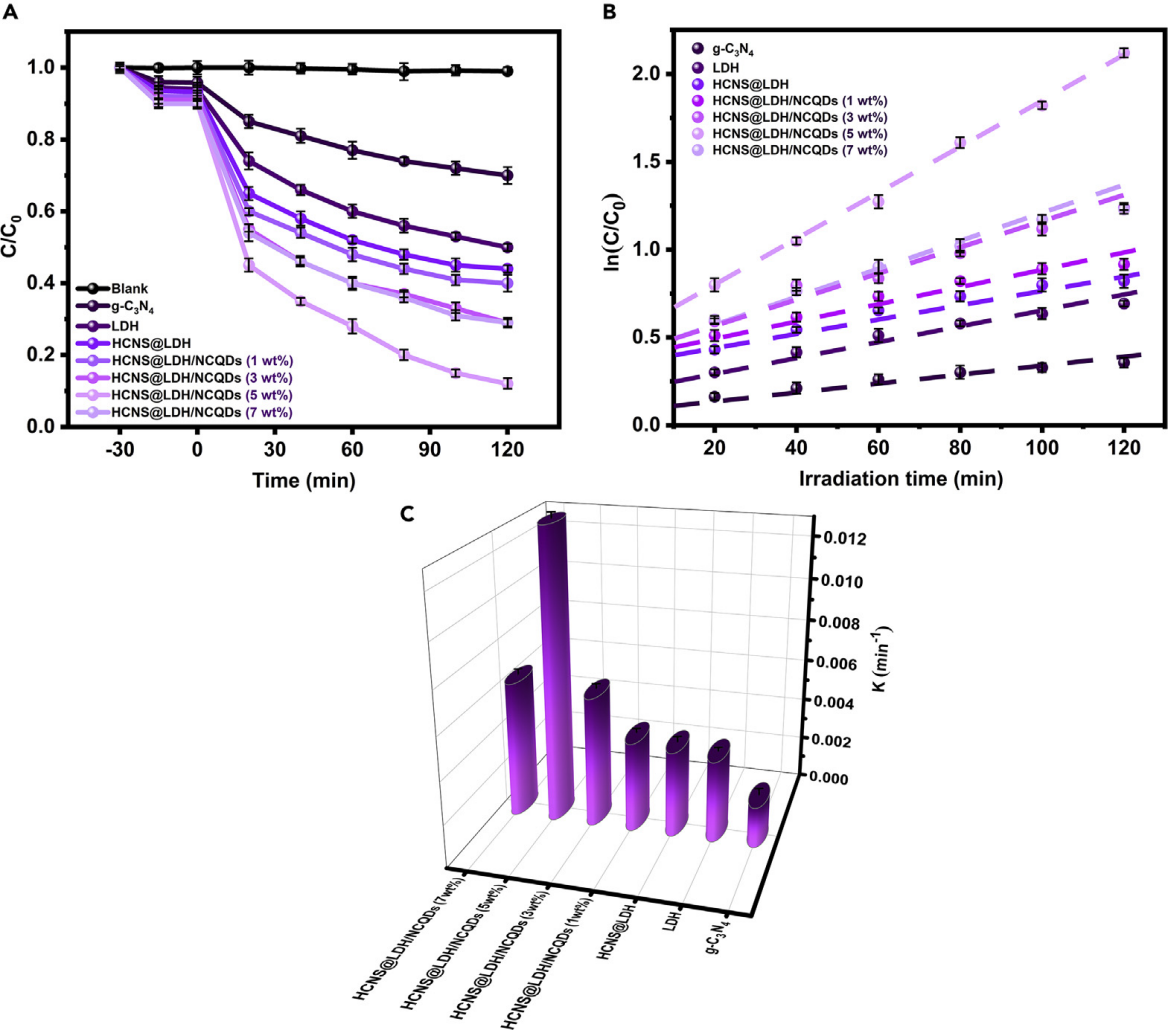
Antibiotic degradation performance (A) Photocatalytic degradation efficiency of TC under visible light irradiation over prepared samples. (B and C) Kinetic curves and reaction rate constant for TC degradation over the as-fabricated photocatalysts.

At first, the effect of NCQDs content on the photocatalytic performance of HCNS@LDH/NCQDs composite was evaluated by the TC degradation under visible light conditions. According to the blank experiment, the degradation of TC without adding any photocatalyst is negligible, which means that a photocatalyst is required for the degradation process. As can be seen in Figure 4A, it is obvious that all the core-shell samples show better photocatalytic performance than pure g-C_3_N_4_ and LDH plates under visible light irradiation. This further proves the high-quality interfacial interactions between HCNS and LDH in the core-shell structures, which improve the charge transfer rate and promote the efficiency of charge separation. Interestingly, the catalytic activity of HCNS@LDH composite was remarkably increased after the loading of NCQDs on the surface of the photocatalyst. Additionally, it is obvious that the amount of NCQDs has an important effect on the TC photodegradation process over the HCNS@LDH/NCQDs composite. With the increase in NCQDs amount from 1 to 5 wt%, the removal efficiency of the composite was increased from 60% to 90% within 120 min. However, the photocatalytic efficiency was noticeably reduced with the further increase in NCQDs up to 7 wt%. This phenomenon could be assigned to the fact that an appropriate amount of NCQDs could act as an electron trap to enhance the separation of charge carriers, but the excess amounts of NCQDs would compete for the photons required for the semiconductors that leads to less photcatalytically active sites. To further understand the degradation dynamic behavior of TC on different samples, the experimental results were assessed by a pseudo-first-order kinetic model with the following equation (Equation 1)^47^:

(Equation 1) $$-\ln\;\left(C_{t}/C_{0}\right)=kt$$

where C_t_ and C_0_ are the concentration at irradiation time t (min) and the initial concentration of TC, respectively, and k represents the reaction rate constant. As shown in Figure 4C, HCNS@LDH/NCQDs (5 wt%) show the highest rate constant among all photocatalysts, giving 7, 4, and 3 times higher than that of pure g-C_3_N_4_, LDH, and HCNS@LDH, respectively. The aforementioned results further reveal that the heterojunction formation between HCNS and LDH nanoplates and the modification of the structure with NCQDs provide a path for the fast transfer of charge carriers. The above results agreed with the previous characterization results, confirming that HCNS@LDH/NCQDs can be a promising candidate for visible-light-driven photocatalytic applications. In addition, the data of our sample (HCNS@LDH/NCQDs) are comparable to or better than those reported by other modified-g-C_3_N_4_ catalysts for photocatalytic TC degradation (see Table S2).

### Photo-excited charge transport dynamics

**Figure 5:**
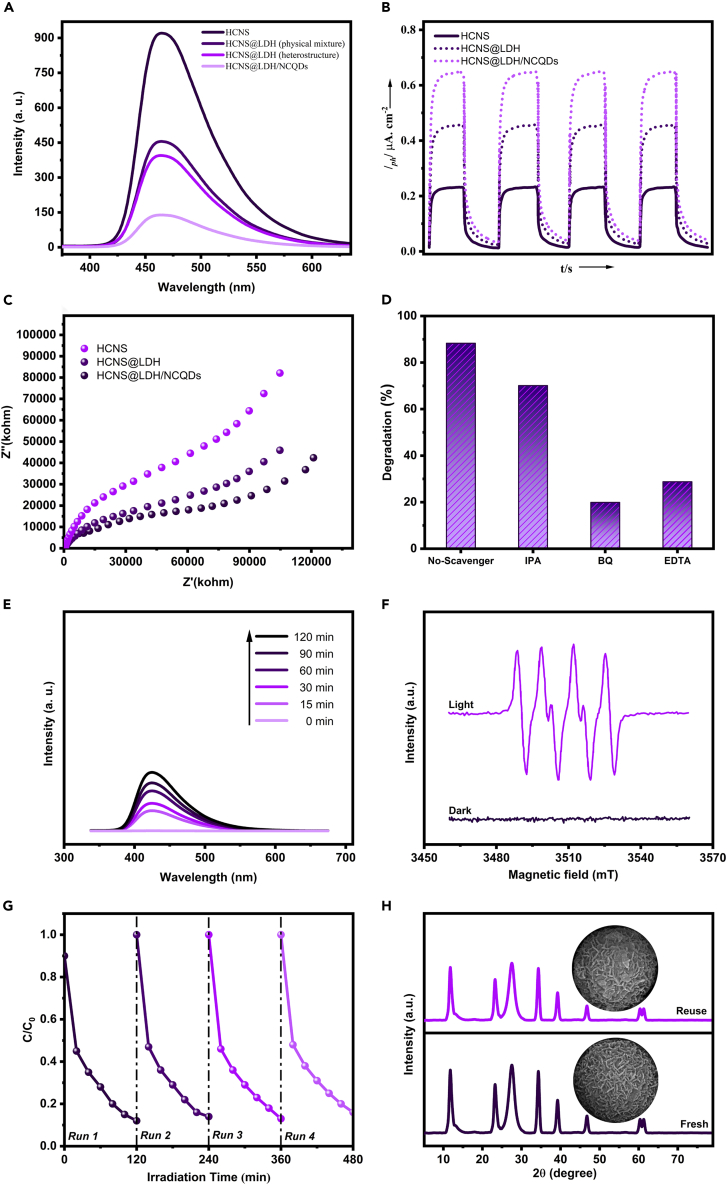
Optical performance and photoelectrochemical performance of prepared samples (A–C) (A) Room temperature PL spectra of prepared samples, (B) transient photocurrent responses, and (C) electrochemical impedance spectroscopy (EIS) changes of HCNS, HCNS@LDH, and HCNS@LDH/NCQDs samples. (D–H) (D) Effects of a series of scavengers (IPA, BQ, and EDTA) on the TC photodegradation over HSNC@LDH/NCQDs photocatalyst, (E) emission intensity of hydroxyterephthalic acid on irradiation of a basic solution of TA over HSNC@LDH/NCQDs at a *λ*_ex_ of 315 nm with different irradiation time; (F) electron spin resonance spectroscopy spectra of HCNS@LDH/NCQDs for DMPO-^·^O_2_^−^; (G) photocatalytic stability tests over HCNS@LDH/NCQDs sample for degradation of TC for four repetitive cycles; (H) XRD patterns and scanning electron microscopy images of HCNS@LDH/NCQDs sample before and after the photocatalytic reaction.

It is known that the separation of photogenerated charges plays a key role in the photocatalytic process.^48^ Steady-state PL spectroscopy was used to evaluate the recombination status of the charge carriers. As shown in Figure 5A, pure HCNS exhibits the highest PL intensity due to the severe recombination rate of charge carriers. An apparent PL quenching is observed for HCNS@LDH, indicating that the recombination rate of electron-hole pairs is effectively suppressed after the heterostructure formation between HCNS and LDH nanoplates. As a comparison, a physical mixture of HCNS and LDH with 20 wt% LDH (LDH content in the HCNS@LDH sample was calculated to be 20 wt% as shown in Figure S2) showed a higher PL emission intensity. This observation implies that the formation of a heterostructure is necessary to promote charge separation efficiency. After the incorporation of NCQDs, the PL intensity exhibited a further decline, signifying the positive effect of NCQDs on restraining the recombination of photo-produced charge carriers. Simultaneously, the transient photocurrent response was tested to evaluate the charge transfer behavior of the catalysts. It could be observed that all samples exhibit repeatable and stable photocurrent profiles to the light illumination during the successive on/off cycles. As depicted in Figure 5B, the HCNS@LDH/NCQDs electrode displays the highest photocurrent density. This result further verifies that the HCNS@LDH/NCQDs sample has the highest charge separation and transfer efficiency. Similarly, the electrochemical impedance spectroscopy measurements in Figure 5C also confirm the above conclusions. Compared with the pure and binary samples, the ternary photocatalyst has a smaller arc radius, illustrating an improved electrical conductivity. The consistency of these analysis results confirms that both the heterojunction formation between HCNS and LDH and the introduction of NDQCs would be beneficial to produce more effective charge carriers in the photocatalytic process.

### Reaction mechanism

To better understand the photocatalytic reaction mechanism of TC degradation, a series of radical scavenging experiments were carried out in the presence of an HSNC@LDH/NCQD photocatalyst. The trapping experiments were the same as the photocatalytic degradation tests, except for the addition of scavengers into the reaction media. Three typical scavengers, including 1,4-benzoquinone (BQ, 0.05 mM), disodium ethylenediaminetetraacetate (EDTA-2Na, 1.3 mM), and isopropanol (IPA, 1 mM) were used as the sacrificial reagents of superoxide radicals (^**·**^O_2_^−^), holes (h^+^), and hydroxyl radicals (^**·**^OH), respectively.^49^ As shown in Figure 5D, the photocatalytic efficiency was remarkably inhibited after the addition of BQ and EDTA-2Na, which verifies the vital role of h^+^ and ^**·**^O_2_^−^ in the photocatalytic process. Unlike the effect of BQ and EDTA-2Na, the addition of IPA has a lower effect on the photocatalytic procedure, indicating that just a few ^**·**^OH radicals took part in the degradation of TC. To further ascertain the involvement of ^**·**^OH in the photocatalytic process, PL spectroscopy was performed using terephthalic acid (TA) as a probe molecule. In this test, TA effectively trapped hydroxyl radicals to produce highly fluorescent 2-hydroxyterephthalic acid, which can be measured by recording the fluorescence emission intensity at 425 nm. Thus, the PL intensity gives indications about the concentration of hydroxyl radicals generated in the photocatalyst aqueous suspension. As can be seen in Figure 5E, there is a slight increase in fluorescence intensity with the increase in irradiation time, indicating that a small quantity of hydroxyl radicals was generated on the surface of the photocatalyst. This result shows that ^**·**^OH is indeed a minor active species in the degradation process, which agrees well with the radical scavenger analysis. To further verify the involvement of ^·^O_2_^−^ in the photocatalytic process, Electron spin resonance spectroscopy (ESR) is employed to probe the generation of superoxide radical species in the photodegradation process, and 5,5-dimethyl-l-pyrroline N-oxide (DMPO) is selected as a trapping agent. As illustrated in Figure 5F, no ESR signals could be observed under dark conditions, but the HCNS@LDH/NCQDs showed a strong DMPO-^·^O_2_^−^ signal under visible light irradiation, which confirmed that a large number of ^·^O_2_^−^ radicals were produced in the photocatalytic process.

Taking inputs from the above photocatalysis experiments and characterization results, a possible mechanism for the photodegradation of TC over the HCNS@LDH/NCQDs composite is proposed and schematically depicted in Figure S3. When the ternary composite is exposed to visible light, the electrons in the valence band (VB) of HCNS and LDH both can be excited to the corresponding conduction band (CB) due to their narrow band gaps, inducing the generation of holes in the VB. Meanwhile, owing to the up-converted PL properties of the NCQDs, light with a long wavelength could be converted to a shorter wavelength that achieved the excitation of the HCNS@LDH system to produce more electron-hole pairs. The VB-XPS spectra were recorded to explore the energy band structure of the semiconductors. As shown in Figure S4, the values of the VB edge levels were determined to be 1.29 and 1.37 eV for HCNS and LDH, respectively, which are close to the previous studies. Based on the equation, E_g_ = E_VB_ − E_CB_, and the UV-Vis results, the CB edges of HCNS and LDH were calculated to be −1.4 and −0.7 eV, respectively. Therefore, the photogenerated electrons on the CB of HCNS could transfer to the VB of LDH, while the VB holes of LDH could migrate to the CB of HCNS, leading to the spatial separation of photoexcited charge carriers. Additionally, due to the excellent electron mobility and affinity, NCQDs can act as the electron mediator to promote the charge transfer process, and the excess electrons also can be transferred to the NCQDs, which further suppresses the recombination of electron-hole pairs. As the E_VB_ of LDH is more negative than the potential of O_2_/^**·**^O_2_^−^ (−0.33 eV), accumulated electrons in the CB of LDH can capture O_2_ to produce ^**·**^O_2_^−^, which played a key role in the photodegradation process as the above experimental results. The photogenerated holes in the VB of HCNS with sufficient potential can be directly used to oxidize the pollutant molecules. Because the redox potential of the top VB of HCNS is smaller than that of ^·^OH/OH^−^ (1.99 eV vs. normal hydrogen electrode [NHE]) and ^·^OH/H_2_O (2.27 eV vs. NHE), it suggests that the photogenerated holes on the surface of HCNS are unable to directly oxidize adsorbed H_2_O or OH^−^ to ^·^OH. It is concluded that the O_2_ adsorbed on the surface of the photocatalyst can capture electrons to produce ^·^O_2_ and/or H_2_O_2_. The produced O_2_^−^ and H_2_O_2_ subsequently converted to ^·^OH with the capture of electrons.^32^^,^^50^

Throughout the above consideration, the reaction steps for the photodegradation process of TC could be expressed as follows:

HCNS@LDH/NCQDs + hυ → HCNS@LDH/NCQDs (e^−^ + h^+^)

O_2_ + e^−^ → ^**·**^O_2_^−^

^**·**^O_2_^−^+ e^−^ + 2H^+^ → H_2_O_2_

H_2_O_2_ + e^−^ → ^**·**^OH

h^+^/^**·**^O_2_^−^/^**·**^OH + TC → degradation product

### The stability evaluation

The photo-stability of the catalyst is another basic characteristic in practical photocatalytic applications. To evaluate the cycling phot-stability of the HCNS@LDH/NCQDs composite, the specimens used in the photocatalytic media were retrieved and used in four repeating cycles for the TC degradation. After each run, the sample was centrifuged, washed with ethanol and ultrapure water, and then dried under a vacuum. As shown in Figure 5G, the photocatalytic activity of the designed photocatalyst had no clear deactivation after four cycles of repeated use, which reveals its high stability with reliable performance in practical applications. Furthermore, the crystallinity and morphology of the cyclic HCNS@LDH/NCQDs photocatalyst were virtually as same as those of the fresh photocatalyst, which indicates the high structural and chemical stability of the catalyst during the photocatalytic reactions (Figure 5H).

### Antibacterial activity

The antibacterial activity of HCNS@LDH/NCQDs (5 wt%) was further studied by inactivating *E. coli* bacteria, as is shown in Figure S5. The experiments were followed by the zones of inhibition through the colony-forming unit determination technique. As observed, the antibacterial activity of the ternary composite under dark conditions was insignificant, while it indicated more antibacterial activity under light exposure. Indeed, the Reactive Oxygen Species (ROS) are generated from active electrons and holes under light irradiation, such as ^.^OH and ^.^O_2_^−^ species have main roles in the destruction mechanism of bacteria.

### Conclusions

In summary, a ternary hierarchical photocatalyst (HCNS@LDH/NCQDs) with a hollow sphere structure has been designed and prepared by a hard-template method combined with a hydrothermal process. By virtue of the special structural features, including hollow porous texture, large surface area, the intimate junction between constituents, and their synergistic effect, the designed nanostructure exhibited significant photocatalytic and antibacterial activities. In particular, the optimized photocatalyst with 5 wt% NCQDs displayed the highest obvious rate constant for the degradation of the TC, which was seven times higher than that of pure g-C_3_N_4_ and four times higher than that of pure LDH. The possible photocatalytic mechanism in the degradation of TC was proposed in detail. Finally, the ternary hybrid exhibited good cycling stability after four cycles, confirming its potential as a visible light photocatalyst for practical application. Furthermore, the optimized sample HCNS@LDH/NCQDs (5 wt% NCQDs) displayed good disinfection activities against *E. coli* bacteria. This study may inspire an effective approach to the synthesis of promising nanostructures with controllable morphologies for visible-light-driven photocatalytic applications.

### Limitations of the study

The *in situ* characterization techniques are necessary for understanding the behaviors of photogenerated charge carriers, the reaction pathways, and mechanisms of hollow structure semiconductors. A major optimization effort is also needed for practical applications.

## STAR★Methods

### Key resources table

**Table:** 

REAGENT or RESOURCE	SOURCE	IDENTIFIER
Tetraethyl orthosilicate	Millipore Sigma	CAS: CAS: 78-10-4
Trimethoxy(octadecyl)silane	Millipore Sigma	CAS: 1341-49-7
Ammonium hydrogen difluoride	Millipore Sigma	CAS: 7647-01-0
Cyanamide	Millipore Sigma	CAS: 420-04-2
Urea	Millipore Sigma	CAS: 57-13-6
Cobalt(II) nitrate hexahydrate	Millipore Sigma	CAS: 10026-22-9
Aluminum nitrate nonahydrate	Millipore Sigma	CAS: 7784-27-2
Ammonium fluoride	Millipore Sigma	CAS: 12125-01-8
Terephthalic acid	Millipore Sigma	CAS: 100-21-0
1,4-Benzoquinone	Millipore Sigma	CAS: 106-51-4
Ethylenediaminetetraacetic acid disodium salt solution	Millipore Sigma	CAS: 139-33-3
Hydrochloric acid	Millipore Sigma	CAS: 3069-42-9
Ammonia solution	Millipore Sigma	CAS: 1336-21-6
2-Propanol	Millipore Sigma	CAS: 67-63-0
Ethanol	Millipore Sigma	CAS: 64-17-5

### Resource availability

#### Lead contact

Further information and requests for resources should be directed to the lead contact, Hossein Vojoudi (hvojoudi@wcupa.edu).

#### Materials availability

Full experimental and electrochemical measurement details can be found in the main text.

### Experimental model and subject details

#### Instrumentation

UV–Vis diffuse reflectance spectroscopy (DRS) was acquired on an Avant spectrophotometer (Avaspec-2048-TEC). The field emission scanning electron microscopy (FESEM) micrographs were visualized with a MIRA3, TESCAN microscope. Transmission electron microscopy (TEM) images were obtained on a Philips CM30 microscope with an accelerating voltage of 150 kV. High-resolution transmission electron microscopy (HRTEM) was performed on a JEOL JEM 2010 - TEM under 220 kV. Powder X-ray diffraction (XRD) was carried out on a Philips diffractometer of X’pert company with monochromatized Cu Kα radiation (λ = 1.5406 Å). The electron spin resource signals were performed to measure the superoxide radicals on a Bruker JES-FA300 spectrometer with spin-trapped reagent 5,5-dimethyl-l-pyrroline N-oxide (DMPO). BET surface area and textural properties were investigated by N_2_ adsorption-desorption with a Micromeritics TriStar II plus apparatus at −196°C. Before measurements, the samples were degassed at 80°C for 12 h under a vacuum. X-ray photoelectron spectroscopy (XPS) measurements were performed using a Thermo Scientific K-Alpha XPS system (Thermo Fisher Scientific, UK) equipped with a micro-focused, monochromatic Al Kα X-ray source (1486.6 eV). Room temperature photoluminescence (PL) spectra of the samples were recorded on the Agilent-G980A instrument. Thermal gravimetric analysis (TGA) was performed on STA BAHR 503 instrument under an N_2_ atmosphere with a heating rate of 10°C min^−1^.

### Method details

#### Synthesis of sacrificial template

A monodisperse core-shell silica template was synthesized by the modified Stöber method.^51^ Typically, 4.3 mL of aqueous ammonia (25 wt.%) and 10 mL of water were added to 74 mL ethanol to form a solution after stirring for 30 min at 30°C. After that, 5.6 mL tetraethyl orthosilicate was added to the above basic ethanol solution with vigorous stirring and was left stationary for one hour to yield monodispersed solid silica spheres. To create a mesoporous shell around the dense cores, a mixture of TEOS (5.2 mL) and C_18_TMOS (2.2 mL) was added dropwise under stirring and kept stationary for 3 h at room temperature. The resulting mixture was centrifuged, dried at 75°C, and calcined at 550°C for 6 h in the air atmosphere with a rate 5°C min^−1^. To better coordinate between the carbon nitride precursor and template, the obtained silica spheres were neutralized through acidification with an HCl solution (1 M, 100 mL) at 80°C for 18 h and then washed with ethanol and dried at 75°C.

#### Synthesis of HCNS

HCNS was synthesized via thermal polymerization, according to a previous report.^33^ In detail, 2 g HCl-treated silica sphere was added to 10 g cyanamide in a 25 mL Pyrex flask and kept under vacuum (0.07 MPa with a vacuum pump) and sonication at 55°C for 4 h. After this, the mixture was stirred at 55°C for 12 h, then centrifuged, dried, ground, and calcined at 550°C under an Ar atmosphere with a ramp 2.3°C min^−1^ for 5 h. The resulting pale-yellow powder was treated with NH_4_HF_2_ (60 mL, 1 M) at room temperature for 24 h to remove the silica template, then centrifuged and washed with ethanol and water every three times. Finally, the yellow HCNS powder was obtained by drying at 70°C for 12 h.

#### Synthesis of NCQDs

2.0 g citric acid and 6.0 g urea were dissolved in 20 mL of distilled water at room temperature. The mixture was transferred to a Teflon-sealed autoclave and heated at 160°C for 6 h. After natural cooling to room temperature, the deep brown solution was centrifuged at 10,000 rpm for 30 min to remove large particles. Finally, the collected supernatant was dried in an oven at 60°C for 16 h to obtain NCQDs.^52^

#### Synthesis of HCNS@LDH-NCDs

100 mg HCNS and the desired amount of NCQDs (1, 3, 5, and 7 wt.% for the weight of HCNS) were dispersed in 20 mL ethanolic solution containing 1 mmol Co(NO_3_)_2_.6H_2_O, 0.33 mmol Al(NO_3_)_3_.9H_2_O, 3.30 mmol urea and, 1.33 mmol NH_4_F using a bath sonicator for 15 min. Then, the total mixture was moved to a Teflon-lined steel autoclave and heated at 100°C for 12 h. After that, the temperature of the autoclave was allowed to cool down naturally, the sample was carefully taken out by centrifugation, washed with ethanol and water several times, and dried at 70°C overnight. For comparison, the HCNS@LDH sample was synthesized under the same conditions without adding the as-prepared NCQDs.

#### Photocatalytic activity evaluation

The photocatalytic activity of the as-prepared samples was evaluated by the degradation of TC under the simulated solar light of a 300 W Xe lamp with a 400 nm cutoff filter. In a typical photodegradation experiment, 100 mg of the photocatalyst was added to 100 mL TC (20 ppm) aqueous solution and stirred in the dark for 20 min to reach the adsorption-desorption equilibrium. Then the suspension was irradiated under the 300 W Xe lamp, and the temperature was kept at room temperature by a circulating water system. At various breaks, 4 mL of the reaction mixture was taken out and centrifuged to remove the photocatalyst particles. The concentrations of TC were monitored by a UV-Vis spectrophotometer (Raleigh UV-1600) at 357 nm. The data in all figures in this study are the average of three replicate results, and the error bars are the standard deviation of the means.

#### Determination of hydroxyl radicals (^·^OH)

The experimental procedure was similar to the photocatalytic degradation but substituting the organic pollutants with the initial concentration of 5 × 10^−4^ mol L^−1^ terephthalic acid (TA) in a dilute sodium hydroxide aqueous solution (2 × 10^−3^ mol L^−1^).^53^ At given time intervals, a portion of the reaction mixture (4 mL) was withdrawn and centrifuged to remove the catalyst particles. After that, the fluorescence emission was measured at 425 nm, using an excitation wavelength of 315 nm.

#### Electrochemical measurements

Electrochemical measurements, including electrochemical impedance spectroscopy (EIS) and transient photocurrent response were operated using a conventional three-electrode system. An Ag/AgCl electrode, platinum plate, and Na_2_SO_4_ solution (0.5 M) were used as the counter electrode, reference electrode, and electrolyte, respectively. To fabricate the working electrode, 5 mg of the photocatalyst was added to 500 μL ethanol and 10 μL Nafion (Sigma Aldrich, 5 wt.%) solution and sonicated for at least 30 min to form a homogeneous slurry. Then, the slurry was uniformly coated on the conductive surface of clean fluorine-doped tin oxide (FTO) glass pieces (1 cm × 1 cm) and allowed to dry naturally. EIS measurements were recorded in the dark circumstance over the frequency range of 1–100 mHz with an alternating voltage of 10 mV, while a 300 W Xe lamp was used as the light source in the photocurrent measurements.

#### Antibacterial activity

The antibacterial activity of HCNS@LDH/NCQDs (5 wt.% NCQDs) was studied by selecting *E. coli* bacteria, which were cultured in broth nutrients kept at 37°C. About 40 mL of fresh PBS solution of the cultured bacteria was mixed with 0.0005 g of the sample and kept under stirring to achieve adsorption-desorption equilibrium under dark conditions.^54^ Finally, the mixture was illuminated under visible light irradiation with continuous stirring, and aliquot samples (0.5 mL) were drawn at an interval of 1 h. The collected samples were diluted and spread on freshly prepared agar plates and incubated at 37°C for two days.

### Quantification and statistical analysis

Details regarding quantification and statistical analysis of photocatalytic data are provided in the results and discussion section under ‘‘Photocatalytic activity evolution’’. All statistical analyses were conducted using OriginLab. Obtained data for catalytic tests is representative for at 3 technical replicates of 3 independent experiments.

## Data Availability

All data supporting this study are available in the Manuscript and supplemental information. This paper does not report original code.
