# Supplementary material for: Facile synthesis of hollow spherical g-C3N4@LDH/NCQDs ternary nanostructure for multifunctional antibacterial and photodegradation activities
Source: iScience. 2023 Feb 16;26(3):106213. doi: 10.1016/j.isci.2023.106213 (PMC9993033; doi:10.1016/j.isci.2023.106213)
Supplement: Document S1. Figures S1–S5 and Tables S1 and S2 [file mmc1.pdf]

iScience, Volume *26*

Supplemental information

Facile synthesis of hollow spherical g-C_3N_4@LDH/NCQDs ternary nanostructure for multifunctional antibacterial and photodegradation activities

Leila Arjomandi-Behzad, Zeinab Alinejad, Mina Ranjbar Zandragh, Amir Golmohamadi, and Hossein Vojoudi

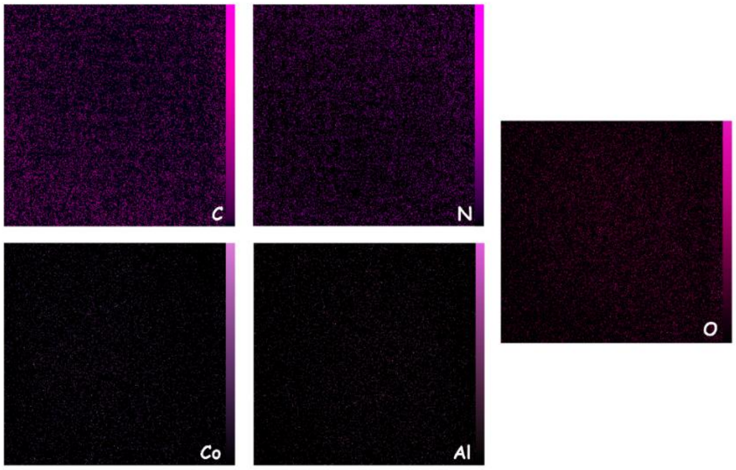

Figure. S1. Elemental mapping images of C, O, N, Co, and Al elements of HCNS@LDH/NCQDs, related to Fig. 1.

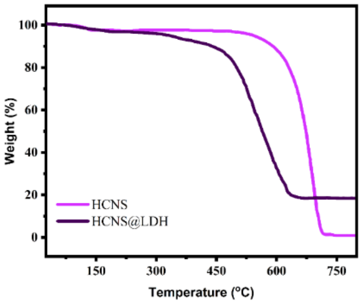

Figure. S2. TGA curve of thermal decomposition of HCNS and HCNS@LDH samples, related to the Fig. 3.

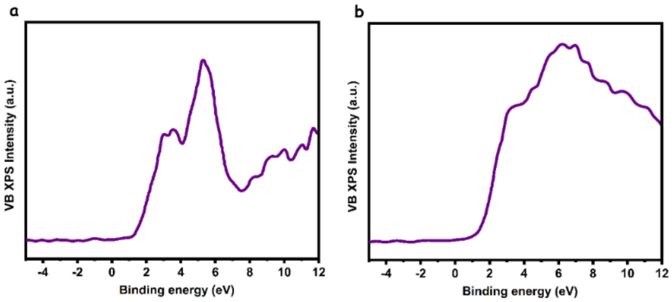

Figure S3. Valance band XPS profiles of CN (a) and LDH (b) photocatalysts, related to reaction Fig. 3 and Fig. 5.

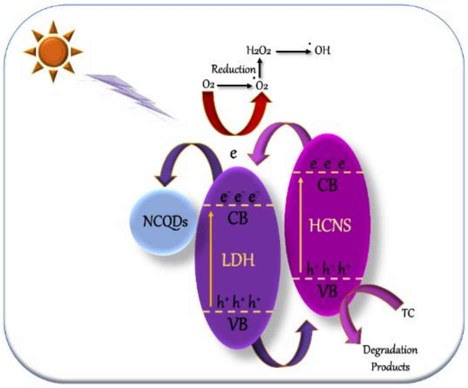

Figure. S4. Schematic diagram of the possible photocatalytic process for degradation of TC over the ternary composite, related to the Fig 4 and Fig. 5.

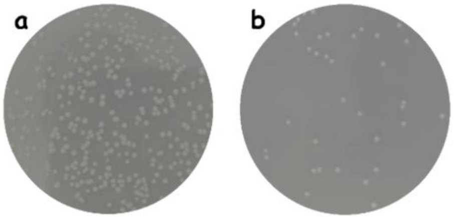

Figure. S5. Antibacterial efficiency of HCNS@LDH/NCQDs (5 wt.%) in the absence (a) and presence (b) of illumination, related to the Fig. 4.

Table S1. Specific surface area, pore volume and pore diameter for different as-prepared materials, related to Fig. 2 and Fig. 3.

Samples	S_{BET} (m^2 g^{-1})	V (cm^3 g^{-1})	D_{BJH} (nm)
HCNS	81	0.32	3.6
LDH	12	0.029	3.2
HCNS@LDH/NCQDs	92	0.38	3.4

Table S2. Comparison of photocatalytic efficiencies of other previously reported g-C_3N_4-based composite photocatalysts for degradation TC in recent years, related to Fig. 4.

Photocatalyst	Light source	Dosage (g. L^{-1})	Irradiation Time (min)	Photocatalytic efficiency (%)	Ref.
Carbon dots/g-C_3N_4	Xe lamp (300 W)	0.4	120	86	[1]
WO_3/g-C_3N4/Bi_2O_3	Xe lamp (300 W)	1	60	80	[2]
TiO_2/g-C_3N_4	Xe lamp (300 W)	0.02	120	85	[3]
Ba/g-C_3N_4	LED (5 W)	1	120	69	[4]
$NiFe_2O_4$/g-C_3N_4	Xe lamp (300 W)	0.02	80	94	[5]
C-doped/g-C_3N_4/WO_3	Xe lamp (500 W)	0.01	60	75	[6]
$ZnSnO_3$/g-C_3N_4	Xe lamp (300 W)	0.01	120	85	[7]
g-C_3N_4 nanoflakes	LED lamp (300 W)	0.02	120	70	[8]
Au-g-C_3N_4-ZnO	Xe lamp (300 W)	0.05	30	74	[9]
PVDF-TiO_2@g-C_3N_4	Xe lamp (300 W)	0.1	300	82	[10]
HCNS@LDH/NCQDs	Xe lamp (300 W)	0.02	120	90	This work

References

1. Shi, W., Yang, S., Sun, H., Wang, J., Lin, X., Guo, F., and Shi, J. (2021). Carbon dots anchored high-crystalline gC_3N_4 as a metal-free composite photocatalyst for boosted photocatalytic degradation of tetracycline under visible light. J. Mater. Sci. 56, 2226–2240.

2. Jiang, L., Yuan, X., Zeng, G., Liang, J., Chen, X., Yu, H., Wang, H., Wu, Z., Zhang, J., and Xiong, T. (2018). In-situ synthesis of direct solid-state dual Z-scheme WO_3/g-C_3N_4/Bi_2O_3 photocatalyst for the degradation of refractory pollutant. Appl. Catal. B Environ. 227, 376–385.

3. Dehkordi, A.B., and Badiei, A. (2022). Insight into the activity of TiO_2@ nitrogen-doped hollow carbon spheres supported on g-C3N4 for robust photocatalytic performance. Chemosphere 288, 132392–132402.

4. Bui, T.S., Bansal, P., Lee, B.-K., Mahvelati-Shamsabadi, T., and Soltani, T. (2020). Facile fabrication of novel Ba-doped g-C_3N_4 photocatalyst with remarkably enhanced photocatalytic activity towards tetracycline elimination under visible-light irradiation. Appl. Surf. Sci. 506, 144184–144195.

5. Liu, S., Zada, A., Yu, X., Liu, F., and Jin, G. (2022). $NiFe_2O_4$/g-C_3N_4 heterostructure with an enhanced ability for photocatalytic degradation of tetracycline hydrochloride and antibacterial performance. Chemosphere 307, 135717–135725.

6. Zhao, C., Ran, F., Dai, L., Li, C., Zheng, C., and Si, C. (2021). Cellulose-assisted construction of high surface area Z-scheme C-doped g-C_3N_4/WO_3 for improved tetracycline degradation. Carbohydr. Polym. 255, 117343–117351.

7. Huang, X., Guo, F., Li, M., Ren, H., Shi, Y., and Chen, L. (2020). Hydrothermal synthesis of ZnSnO3 nanoparticles decorated on g-C_3N_4 nanosheets for accelerated photocatalytic degradation of tetracycline under the visible-light irradiation. Sep. Purif. Technol. 230, 115854–115861.

8. Hu, C., Liu, Z.-T., Yang, P.-C., Ding, Y.-X., Lin, K.-Y.A., and Nguyen, B.-S. (2021). Self-assembly L-cysteine based 2D g-C_3N_4 nanoflakes for light-dependent degradation of rhodamine B and tetracycline through photocatalysis. J. Taiwan Inst. Chem. Eng. 123, 219–227.

9. Huang, L., Bao, D., Li, J., Jiang, X., and Sun, X. (2021). Construction of Au modified direct Z-scheme g-C_3N_4/defective ZnO heterostructure with stable high-performance for tetracycline degradation. Appl. Surf. Sci. 555, 149696–149706.

10. Zheng, X., Liu, Y., Liu, X., Li, Q., and Zheng, Y. (2021). A novel PVDF-TiO_2@ g-C_3N_4 composite electrospun fiber for efficient photocatalytic degradation of tetracycline under visible light irradiation. Ecotoxicol. Environ. Saf. 210, 111866–111875.
